# Genome-Wide Association Study Reveals Novel Quantitative Trait Loci Associated with Resistance to Multiple Leaf Spot Diseases of Spring Wheat

**DOI:** 10.1371/journal.pone.0108179

**Published:** 2014-09-30

**Authors:** Suraj Gurung, Sujan Mamidi, J. Michael Bonman, Mai Xiong, Gina Brown-Guedira, Tika B. Adhikari

**Affiliations:** 1 Department of Plant Pathology, University of California Davis, United States Department of Agriculture-Agricultural Research Service (USDA-ARS), Salinas, California, United States of America; 2 Department of Plant Sciences, North Dakota State University, Fargo, North Dakota, United States of America; 3 USDA-ARS, Small Grains and Potato Germplasm Research Unit, Aberdeen, Idaho, United States of America; 4 USDA-ARS, Plant Science Research Unit, Department of Crop Science, North Carolina State University, Raleigh, North Carolina, United States of America; 5 Center for Integrated Pest Management and Department of Plant Pathology, North Carolina State University, Raleigh, North Carolina, United States of America; China Agricultural University, China

## Abstract

Accelerated wheat development and deployment of high-yielding, climate resilient, and disease resistant cultivars can contribute to enhanced food security and sustainable intensification. To facilitate gene discovery, we assembled an association mapping panel of 528 spring wheat landraces of diverse geographic origin for a genome-wide association study (GWAS). All accessions were genotyped using an Illumina Infinium 9K wheat single nucleotide polymorphism (SNP) chip and 4781 polymorphic SNPs were used for analysis. To identify loci underlying resistance to the major leaf spot diseases and to better understand the genomic patterns, we quantified population structure, allelic diversity, and linkage disequilibrium. Our results showed 32 loci were significantly associated with resistance to the major leaf spot diseases. Further analysis identified QTL effective against major leaf spot diseases of wheat which appeared to be novel and others that were previously identified by association analysis using Diversity Arrays Technology (DArT) and bi-parental mapping. In addition, several identified SNPs co-localized with genes that have been implicated in plant disease resistance. Future work could aim to select the putative novel loci and pyramid them in locally adapted wheat cultivars to develop broad-spectrum resistance to multiple leaf spot diseases of wheat via marker-assisted selection (MAS).

## Introduction

Wheat (*Triticum aestivum* L. subsp. *aestivum*) is the major staple for more than 35% of the world's population [1]. In particular, the demand for wheat has been always high in the developing world. To meet the projected global food demand by 2050 and alleviate poverty [2], [3], the pace of wheat improvement must accelerate. However, wheat production faces numerous threats, especially climatic changes and onset of severe plant disease epidemics, which significantly reduce both yield and grain quality [2], [3], [4], [5]. Among plant diseases, bacterial leaf streak (BLS) [caused by *Xanthomonas translucens* pv. *Undulosa* [6], Tan spot [*Pyrenophora tritici-repentis* (PTR) (Died.) Drechs.], Spot blotch (SB) [*Cochliobolus sativus* (Ito & Kuribayashi) Drechsler ex Dastur)], Stagonospora nodorum blotch (SNB) [*Phaeosphaeria nodorum* (E. Müller) Hedjaroude], and Septoria tritici blotch (STB) [*Zymoseptoria tritici* (Desm.) Quaedvlieg & Crous, *comb. nov.*], are the most devastating leaf spot diseases of wheat worldwide [7], [8], [9], [10], [11]. These diseases can cause up to 50% yield reduction under conditions conducive to disease development [12], [13].

Development and deployment of host plant resistance is the most practical approach to manage leaf spot diseases of wheat. Breeding for disease resistance in plants is often difficult, though, especially when resistance to several diseases is needed. In addition, resistance can be inherited both qualitatively and quantitatively as is the case in wheat for Tan spot [14], [15], [16], [17], SB [18], [19], SNB [20], [21], [22], and STB [9], [10], [23], [24], [25] diseases and resistance genes can lose their effectiveness over time due to changes in pathogen populations. Given these challenges, finding new resistance genes and the use of marker-assisted selection (MAS) would aid breeding for disease resistance in wheat. Most wheat breeding programs still focus on linkage mapping or bi-parental mapping to identify important qualitative and quantitative loci responsible for resistance to leaf spot diseases of wheat and markers associated with disease resistance [9], [10], [16], [18], [19], [25]. Using linkage or bi-parental mapping, several quantitative trait loci (QTL) responsible for resistance to PTR, SB, SNB and STB have been identified. For example, using bi-parental mapping QTL responsible for resistance to PTR, *QTsc.ndsu1A*, was found linked with marker *Gli1* [26]. Likewise, Faris and Friesen [27] detected several other PTR non-race specific QTL, such as *QTs.fcu-1BS* on chromosome 1B between markers *Xgdm33* and *Xgdm*125 (10 cM interval), *QTs.fcu-3BL* on chromosome 3B between markers *Xbarc248* and *Xfcp83* (128 cM interval), and *QTs.fcu-3BS* on chromosome 3B between makers Xfcp311 and *Xfcp114* (55 cM interval). Other five QTL, *QTs.fcu-2AS* on chromosome 2A between markers *Xgwm515* and *Xfcp526* (24.2 cM interval), *QTs.fcu-4AL* on chromosome 4A between markers *Xbarc236* and *Xgwm644* (15.8 cM interval), *QTs.fcu-5AL* on chromosome 5A between markers *Xbarc1061* and *Xcfa2185* (25.4 cM interval), *QTs.fcu-5BL.1* on chromosome 5B between markers *Xbarc138* and *Xgwm*260 (35.3 cM interval), and *QTs.fcu-5Bl.2* on chromosome 5B between markers *Xfcp615* and *Xbarc142* (40.5 cM interval) were reported by Chu et al. [15]. Similarly, Chu et al. [16] reported several other PTR resistance QTL: *QTs.fcu-3A* on chromosome 3A between markers *Xbarc321* and *Xwmc11* (3.7 cM), *QTs.fcu-3B* on chromosome 3B between markers *Xwmc231* and *Xwmc762* (16.7 cM), *QTs.fcu-5A* on chromosome 5A between markers *Xgwm425* and *Xgwm6.1* (64.9 cM interval), *QTs.fcu-5A.2* on chromosome 5A between markers *Xwmc110* and *Xgwm595* (25.1 cM interval), and *QTs.fcu-7B* on chromosome 7B between markers *Xwmc276* and *Xbarc182* (13.1 cM interval). Kumar et al. [18] detected four QTL resistance to SB: *QSb.bhu-2A* on chromosome 2A between markers *Xbarc353* and *Xgwm445* (37.4 cM interval), *QSb.bhu-5B* on chromosome 5B between markers *Xgwm067* and *Xgwm371* (13.2 cM interval), *QSb.bhu-2B* on chromosome 2B between markers *Xgwm148* and *Xgwm374* (15.0 cM interval), and *QSb.bhu-6D* on chromosome 6D between markers *Xbarc175* and *Xgwm732* (30.1 cM interval). Similarly, four SB resistant QTLs, *QSb.bhu-2A* on chromosome 2A between markers *Xgwm425* and *Xbarc159* (8 cM interval), *QSb.bhu-5B* on chromosome 5B between markers *Xgwm067* and *Xgwm213* (9 cM interval), *QSb.bhu-2B* on chromosome 2B between markers *Xgwm148* and *Xbarc91* (21.2 cM interval), and *QSb.bhu-7D* on chromosome 7D between markers *Xgwm111* and *Xgwm1168* (3 cM interval) were detected via linkage mapping [19]. For SNB disease, a toxin sensitivity locus *Snn1* was associated with a marker *XksuD14* (4.7 cM far from the *Snn1* locus) using biparental mapping [28]. Abeysekara et al. [29] reported another toxin sensitivity locus, *Snn4*, on chromosome 1A between markers *XBG262267* and *Xksum182.1* (<1 cM interval), and another locus linked with marker *XBF293121* on chromosome 7A. The SNB flag leaf resistance QTL denoted as *QSn1.daw-2A* was detected on chromosome 2A between markers *wPt2448* and *wPt7056* (24.4 cM interval) during 2004 and between markers *gwm614a* and *wPt9432* (29.5 cM interval) during 2005 [30]. Two other QTL, *QSn1.daw-4B* and *QSn1.daw-5B*, were flanked by markers *barc0163* and *wPt0391* (29.6 cM interval) and *wPt4628* and *wPt1733* (32.2 cM interval), respectively, using biparental mapping [30]. At least, 17 STB resistance genes and QTL have been reported using biparental mapping. Among these, two STB resistance QTL, *QStb.risø-3A.2* and *QStb.risø-6B.2*, were reported at supporting intervals 55–61 and 82–90 cM on chromosomes 3A and 6B [31]. Similarly, the STB resistance locus *Stb16q* was flanked by markers *Xbarc12*5 and *Xbarc128* (16.6 cM interval), and another locus *Stb17* was flanked by markers *Xgwm617* and *Xhbg247* (26.8 cM interval) [32]. Chartrain et al. [33] detected the STB-resistance gene *Stb9* located between markers *Xfbb226* (3·6 cM) and *XksuF1b* (9 cM) on the long arm of chromosome 2B. Although the biparental mapping approach has been useful for detecting major genes and QTL, this technique is time-consuming and labor intensive. In addition, the relatively few recombination events in bi-parental mapping populations has limited the identification of closely linked markers useful for MAS due to long linkage blocks [34]. Recently, genomic analysis of diverse populations is increasingly being used to uncover the genetic basis of complex traits of crops [35]. For example, a genome-wide association study (GWAS) of 358 European winter wheats detected several previously identified major genes (*Tsn1*, *tsn2*, *tsn5*, *Tsc2*, *Tsr6*) and several other QTL [36]. Using a GWAS approach, Miedaner et al. [37] discovered eight single nucleotide polymorphism (SNP) markers that were significantly associated with resistance to STB in European wheat lines. The GWAS approach was able to identify regions with STB resistance that had been previously identified using linkage mapping [38]. Additionally,GWAS has been also useful in elucidating the genetic basis of agronomic and agro-climatic traits in maize [39], barley [40], tomato [41], and rice [42].

We previously identified a set of Diversity Arrays Technology (DArT) loci associated with resistance to BLS, PTR races 1 and 5, SB, and SNB [43], [44], [45]. A major limitation using DArT markers is that these markers are not uniformly distributed across wheat genome. In addition, only relatively few polymorphic markers were identified that could be used for analysis. More recently, genome-wide SNP markers have been used to uncover multiple targets for wheat improvement [46]. We hypothesize that these newly developed SNPs would be associated with loci conferring resistance to multiple leaf spot diseases of wheat and could be used to validate the QTL identified previously using DArT markers [43], [44], [45]. Using sequence data associated with the significant SNPs, it is now possible to postulate the potential biological function related to resistance. To discover new allelic diversity and loci underlying resistance to major leaf spot diseases, and accelerate MAS, we characterized 528 diverse spring wheat accessions from the USDA-ARS National Small Grains Collection (NSGC) using 9K SNP wheat chip and GWAS analysis.

## Materials and Methods

### Association mapping panel

The association mapping panel utilized for GWAS consisted of 528 hexaploid spring wheat accessions. These accessions were locally-grown landraces originated from 55 countries in six continents and held by the NSGC in Aberdeen, ID. Where necessary, accessions were advanced by single plant selection. To identify QTL associated with STB resistance, we performed two independent experiments in controlled growth chambers as described previously [15]. A highly aggressive test isolate of *Zymoseptoria tritici* (Ma04-9-4) from North Dakota [3], [4], [47] was selected and inoculum was prepared as described previously [47]. Seven–week-old plants were spray-inoculated (20 ml inoculum per pot) with a hand sprayer and immediately transferred into a mist chamber with 100% relative humidity at 24°C for 72 to 96 h. Test plants then were transferred to a growth chamber programmed for a 22/18°C diurnal temperature and a 16-h photoperiod. Flag leaves were assessed 21 to 28 days post inoculation based on percentage of leaf area of necrotic lesions containing pycnidia [4]. The disease severity rating scale was from 0 to 100%, where the accessions with scores ≤30% severity were classified as resistant and those with scores>30% were classified as susceptible. Analysis of variance (ANOVA) was conducted using Statistical Analysis System (SAS) software version 9.3 (SAS Institute, Cary, NC). To identify novel QTL, the STB phenotypic data from this study, and the BLS, PTR races 1 and 5, SB and SNB phenotypic data from the previous studies [43], [44], [45] were utilized for GWAS and performed individually.

### SNP marker data

Briefly, the 528 spring wheat landraces were genotyped at the Regional Genotyping Laboratory, USDA-ARS, Fargo, North Dakota using the Illumina iSelect beadchip assay for wheat having 9,000 SNPs. To avoid monomorphic and low-quality SNPs, data was sorted using Genome Studio software [46]. Nearly 5,634 informative SNPs were selected and used for GWAS. Missing data were imputed using the FastPHASE [48] with default settings. Markers with minor allele frequencies (MAF) less than 0.05 were removed from the data set in subsequent analysis, since the power of association in these alleles were low [49].

### Population structure and relatedness

Population structure was analyzed using two methods. The principal components (PC) were estimated in SAS 9.3 using the Princomp procedure. The principal components were further used for GWAS. The population structure was also estimated using STRUCTURE.2.3.4 [50]. The admixture model with a burn-in was 100,000 and 500,000 iterations was used for each run. The subpopulations tested range from 1 to 15 and five runs for each K value were performed. The optimum number of subpopulations was determined by the Wilcoxon two sample test as described by Rosenberg et al. [51] and Wang et al. [52]. The Delta K approach used structure harvester [53] and the Wilcoxon test compared the posterior probabilities of two successive sub-populations (k1 vs. k2, k2 vs. k3, k3 vs. k4, and so on) using the NPAR1WAY procedure in SAS. The smaller k value in a pairwise comparison for the first non-significant Wilcoxon test was chosen as the best number of subpopulations [54], [55], [56]. These results were further used to interpret the geographic distribution of the landraces.

### Genome-Wide association analysis

We employed four regression models: Naïve, PC, Kinship, and PC+Kinship. Among these, the Naïve model that did not account for population structure and relatedness and the regression model with only PC were analyzed in SAS 9.3. Models with only kinship and a combination of both PC and kinship were analyzed in Gemma 0.92 [33]. The number of PCs explaining 50% of the cumulative variation were used in the regression model to control for population structure and the kinship matrix estimated as a center matrix using Gemma 0.92 [57] was used to control for population relatedness. The underlying regression equation for the association mapping analysis is y = Xα+Pβ+Iν+ε where, y is a vector of phenotypic values, α is the fixed effect for the candidate marker, β is a vector of fixed effects regarding population structure, X is the vector of genotypes at the candidate marker. P is a matrix of the principal components, ν is a vector of the random effects pertaining to co-ancestry; I, is an identity matrix, and ε is a vector of residuals. The variances of the random effects are assumed to be Var(ν) = 2KVg and Var(ε) = IV_R_, where K is the kinship matrix that defines the degree of genetic covariance between a pair of individuals, Vg is the genetic variance and V_R_ the residual variance [58]. Among the four models for each trait, a best model was selected based on the smallest Mean Square Difference (MSD) between the observed and expected p-values [54], since the random marker p-values follow a uniform distribution [59].

### Marker-Trait associations

Association between SNPs and disease resistance traits were considered significant if the p-value was ≤0.001 [60], [61]. To detect significant markers for each trait, the phenotypic variation (R^2^) was calculated using a simple regression equation implemented in General Linear Model (GLM) procedure in SAS 9.3. The least squares means of the alleles of significant markers were estimated using the GLM procedures with six principal components as covariates in the model. In addition, stepwise regression implemented in the SAS REG procedure was used to estimate the combined variation explained by the markers. A significant p-value of 0.05 was necessary for both marker and model for stepwise inclusion of the marker in REG procedure in SAS 9.3. This approach identified the major markers within a QTL excluding markers in LD of the QTL. In addition, this approach includes only the markers from major QTL masking the effects of minor QTL and has the advantage of considering the correlations and interactions between the QTL [62]. This subset explains the most phenotypic variation similar to variation explained by all markers together. A subset of markers is also more easily used for MAS compared to the entire set of markers. The adjusted consensus map developed using seven parental crosses that has 7,497 markers mapped on 21 chromosomes (represented as 25 LG) [46], were used to position the QTL on the wheat genome.

### SNP marker annotations

The sequences of the significant markers available for 9K SNP wheat chip [46] were blasted against gene models of *Brachypodium distachyon* [63], *Oryza sativa* [64], and *Sorghum bicolor* [65] that are available at phytozome.net. The search was limited to the top hit with an E-value cut off of at least 1E-10. Further, we determined if the significant marker was in the coding or non-coding region. If the marker was in coding region, the substitution was designated as synonymous (no change in amino acid) or non-synonymous substitution (change in amino acid).

### Allelic combinations

Allelic combination refers to the combination of the marker alleles that effect the changes in phenotype. To discover allelic combinations, we employed SNPs in stepwise regression and calculated the mean and standard deviation of the phenotype. Based on the cut-off scale for resistance and susceptibility, allelic combinations were further used to identify resistant sources.

### Genome-wide linkage disequilibrium

Linkage disequilibrium (LD) is the square of the correlation coefficient (r^2^) between markers. To investigate the extent of LD across the wheat genome and the markers that have a position on the consensus map [46], r^2^ between intra chromosomal SNP markers was estimated using SAS 9.3. We plotted the intra-chromosomal r^2^ values against the genetic distance, using a non-linear regression in SAS [66]. The distance at which the LD decays to 0.7 was considered as the critical distance up to which a QTL region extends.

## Results

### Phenotypic diversity

Association mapping panel exhibited substantial phenotypic diversity for all leaf spot diseases investigated (Table 1). The distribution of phenotypes ranged from susceptible to resistant across the 528 accessions (Table S1). ANOVA revealed that the interactions between the two STB experiments were not significant (*p*≤0.05), suggesting that the results of both experiments were independent. The Bartlett's chi-square (χ^2^) value was 5.1 and the associated *p* value with 1 degree of freedom was 0.04. Therefore, data from homogenous experiments were pooled and used for GWAS. Similar procedures were used for BLS, PTR race 1, PTR race 5, SB, and SNB [25], [26], [27]. Pair-wise comparison of the Pearson correlation coefficient values across diseases were significant (*p*<0.05) except for PTR race 1 and SNB, SNB and BLS, and SNB and SB (Table 2).

**Table 1:** Statistical properties of major leaf spot diseases analyzed in this study.

Disease^A^	Mean	Standard deviation	Minimum	Maximum	p-value of Ks test^B^	Evaluated score range	Cutoff score for resistant
BLS	3.51	1.11	0.63	5.00	<0.01	0 to 6	2
PTR1	3.47	0.89	0.44	5.00	<0.01	1 to 5	2
PTR5	2.64	0.76	1.00	4.50	<0.01	1 to 5	2
SNB	2.50	0.95	0.00	5.00	0.048	0 to 5	3
SB	4.86	0.87	3.00	8.00	<0.01	1 to 9	4
STB	37.94	28.88	0.25	100.00	<0.01	0 to 100%	30%

A BLS  =  Bacterial leaf streak, PTR 1  =  *Pyrenophora tritici-repentis* race 1, PTR 5  =  *Pyrenophora tritici-repentis* race 5, SB  =  Spot blotch, SNB  =  Stagonospora nodorum blotch, and STB  =  Septoria tritici blotch, respectively.

B Kolmogorov–Smirnov test.

**Table 2:** The relationships between the Pearson correlation coefficient values (lower diagonal) and the p-values (upper diagonal) among major leaf spot diseases of spring wheat analyzed.

Disease^A^	BLS	PTR1	PTR5	SNB	SB	STB
BLS		<.0001	0.0294	0.1875	<.0001	0.3802
PTR1	0.219		<.0001	0.1817	<.0001	<.0001
PTR5	0.094	0.306		0.0005	0.0015	<.0001
SNB	0.056	0.057	−0.150		0.1164	0.0205
SB	0.183	0.178	0.136	−0.067		<.0001
STB	−0.037	0.178	0.229	−0.099	0.183	

A BLS  =  Bacterial leaf streak, PTR 1  =  *Pyrenophora tritici-repentis* race 1, PTR 5  =  *Pyrenophora tritici-repentis* race 5, SB  =  Spot blotch, SNB  =  Stagonospora nodorum blotch, and STB  =  Septoria tritici blotch, respectively.

### SNP marker data

**Figure 1:**
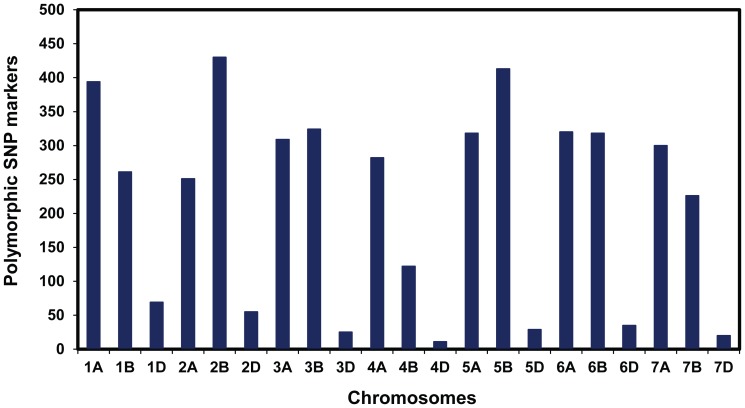
Distribution of polymorphic single nucleotide polymorphism (SNP) markers throughout the wheat chromosomes.

Of the 5,634 SNPs obtained from the 9K SNP wheat chip, 80% had known chromosomal locations [46]. Heterozygotes accounted for 0.1% of the SNPs and were converted to missing data to estimate for sequencing errors. Approximately, 1% of the missing data was imputed using a likelihood approach. In total, 4,781 SNPs with minor allele frequencies greater than 5% were used for subsequent analysis. Of the total 4781 polymorphic markers, 94.37% (4512 SNPs) had known chromosome positions. The mapped markers were not evenly distributed across wheat genome. Our analysis revealed 45.47%, 43.79% and 5.10% of the SNPs were distributed on wheat genome A, B, and D, respectively (Figure 1). Nearly 430 (8.99%) polymorphic SNPs were on chromosome 2B. In contrast, only 11 (0.23% of the total) polymorphic SNPs were detected on chromosome 4D.

### Population structure

**Figure 2:**
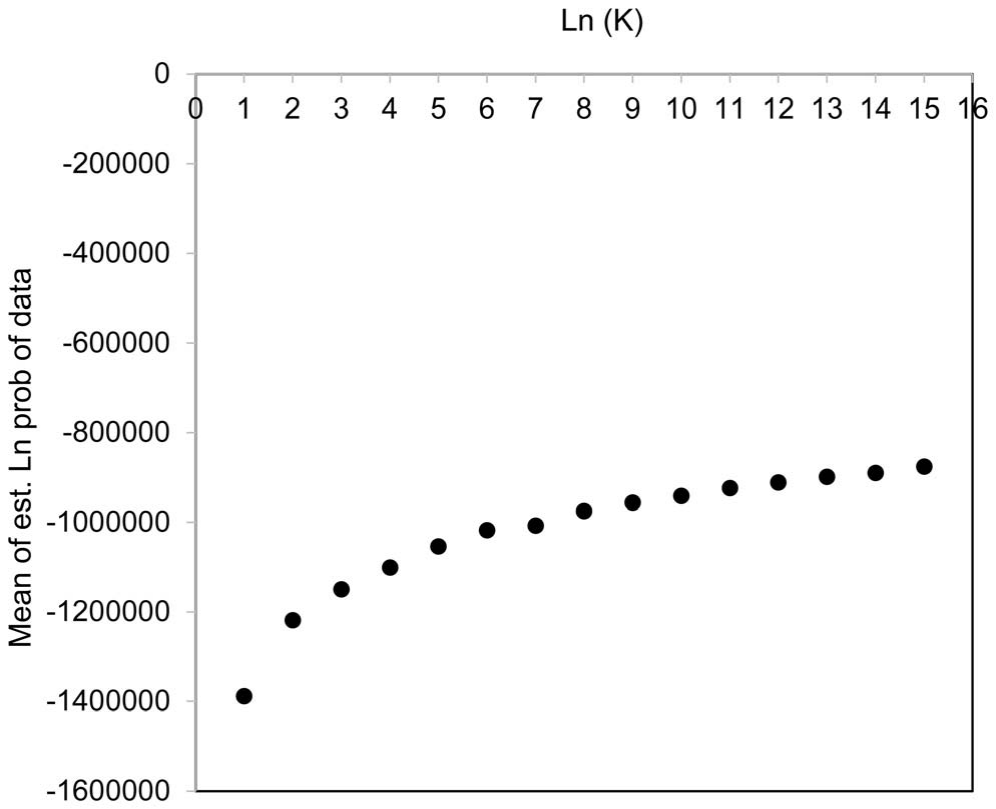
Analysis of population structure using Wilcoxon test.

The Bayesian based clustering approach implemented in STRUCTURE revealed the presence of six subpopulations evaluated using the Wilcoxon test (Figure 2). A majority of the individuals have a membership coefficient (q^i^)>0.7 to be assigned to a subpopulation revealing a strong population structure among individuals with little admixture [67], [68]. However, these populations could not be assigned based on the geographic regions, because each of these populations identified from STRUCTURE analysis had wheat accessions from Africa, Asia, Europe and the Americas.

Based on the MSD for the four regression models tested, the regression model that has only Kinship was considered best for PTR race 1, PTR race 5 and SNB (Table 3; Figure S1). Similarly, mixed model containing PC and Kinship was considered best for BLS, SB and STB (Figure S1; Table 3).

**Table 3:** Mean square difference for four models used to identify the best regression model to discover single nucleotide polymorphisms (SNPs) and leaf spot disease resistance trait - marker associations.

Trait^A^	Naïve	PC	Kinship	PC + kinship
BLS	0.1668	0.0182	1.52E-04	***5.70E-05*** B
PTR1	0.0445	0.01	***4.07E-05***	1.17E-04
PTR5	0.0504	0.0146	***1.58E-05***	2.78E-04
SB	0.0948	0.0071	0.0013	***2.71E-04***
SNB	0.0641	0.0071	***1.62E-05***	5.18E-04
STB	0.0535	0.0384	1.54E-04	***6.85E-05***

A BLS  =  Bacterial leaf streak, PTR 1  =  *Pyrenophora tritici-repentis* race 1, PTR 5  =  *Pyrenophora tritici-repentis* race 5, SB  =  Spot blotch, SNB  =  Stagonospora nodorum blotch, and STB  =  Septoria tritici blotch, respectively.

B Bold and italicized numbers indicate lowest mean square deviation (MSD) and best fit model for each disease trait.

### Marker -Trait associations and annotations

**Figure 3:**
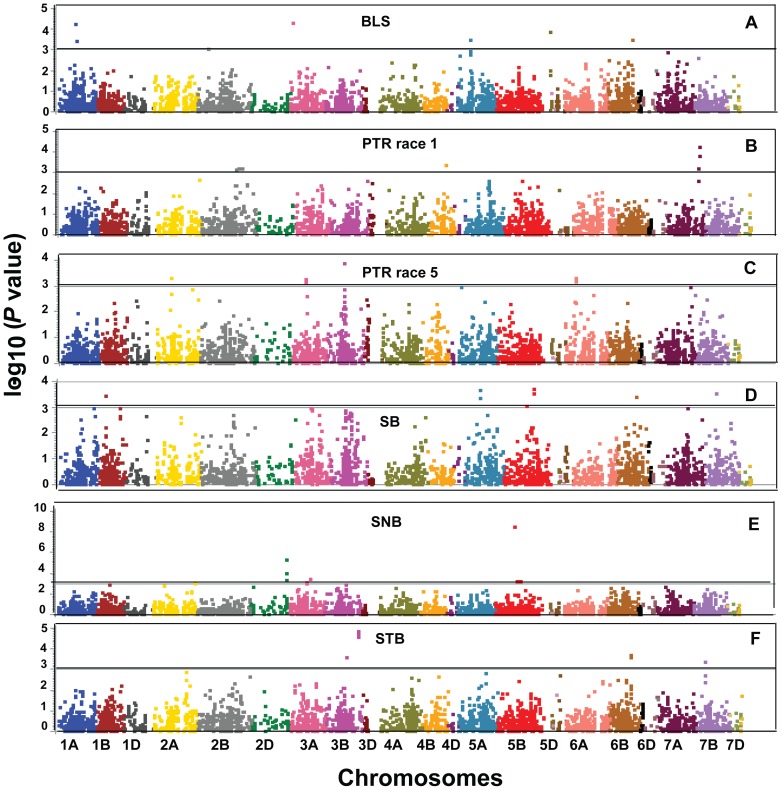
Manhattan plots for major leaf spot diseases, (A) Bacterial leaf streak (BLS), (B) *Pyrenophora tritici-repentis race* 1 (PTR 1) (C) *Pyrenophora tritici-repentis* race 5 (PTR 5), (D) Spot blotch (SB), (E) *Stagonospora nodorum* blotch (SNB), (F) *Septoria tritici* blotch (STB) and significant association signals. *P* values are shown on a log_10_ scale. The marker is considered significant if p value <0.001.

Eight SNP markers were significantly (p<0.001) associated with resistance to BLS and detected on chromosomes 1A, 2B, 3A, 5A, 5D and 6B (Table 4, Figure 3). The phenotypic variation ranged from 1.9 to 7.6% (Table 4). Of the eight significant SNPs identified, five were associated with a gene model (Table 5). Among five changes, three were non-synonymous on chromosome 5A, 5D and 6B and two changes were synonymous on chromosome 1A (Table 5). Of the eight significant SNPs identified, four fit into a stepwise regression and explained 14.3% of the phenotypic variation (Table 6). These SNPs belong to four QTL regions on chromosomes 1A, 5A, 5D, and 6B.

**Table 4:** Significant associations between single nucleotide polymorphism (SNP) markers and resistance to major leaf spot diseases detected in the 528 spring wheat landraces.

Disease^A^	Marker	Chromosome	position	Log_10_ (p-value)	Allele 1	No. of genotypes	Mean	Allele 2	No. of genotypes	Mean	MAF	R-square (as %)^C^	Included in stepwise regression
BLS	wsnp_Ex_c44263_50363536	***1A*** B	82.03	4.19	C	363	2.37	T	180	2.74	33.15	2.51	
BLS	wsnp_Ex_c8588_14419007	***1A***	85.09	3.4	C	199	2.69	T	344	2.38	36.65	2.33	
BLS	wsnp_Ku_c23926_33870364	***1A***	85.09	3.4	C	344	2.38	T	199	2.69	36.65	2.33	
BLS	wsnp_Ex_c10596_17293363	2B	53.01	3.01	C	198	2.28	T	345	2.61	36.46	2.90	Yes
BLS	wsnp_CAP11_rep_c4157_1965583	3A	12.84	4.25	C	264	2.32	T	279	2.65	48.62	4.64	Yes
BLS	wsnp_Ex_c5998_10513766	5A	64.81	3.42	C	131	2.16	T	412	2.60	24.13	7.60	Yes
BLS	wsnp_Ex_rep_c67164_65655648	5D1cult	42.45	3.82	C	268	2.66	A	275	2.33	49.36	6.31	Yes
BLS	wsnp_Ku_c11846_19263340	***6B***	120.91	3.44	G	453	2.53	A	90	2.28	16.57	1.88	
PTR1	wsnp_BF473744B_Ta_2_2	***2B***	169.50	3.12	C	389	3.59	A	154	3.16	28.36	4.39	Yes
PTR1	wsnp_Ex_rep_c67561_66189356	***2B***	185.08	3.18	G	372	3.61	A	171	3.17	31.49	4.74	
PTR1	wsnp_Ex_c19772_28771627	***2B***	203.24	3.19	C	146	3.75	T	397	3.37	26.89	2.56	Yes
PTR1	wsnp_Ex_rep_c67159_65649966	4B	85.23	3.33	C	447	3.53	T	96	3.18	17.68	1.03	Yes
PTR1	wsnp_Ex_c9971_16412345	7A	154.22	3.15	C	108	3.15	T	435	3.55	19.89	5.52	Yes
PTR1	wsnp_Ex_c9971_16412270	7A	159.51	4.22	C	489	3.54	T	54	2.87	9.94	6.46	Yes
PTR1	wsnp_Ex_c9971_16412758	7A	159.51	3.8	G	39	2.85	A	504	3.52	7.18	5.33	
PTR1	wsnp_Ku_c26118_36079171	7A	159.51	4.22	G	54	2.87	A	489	3.54	9.94	6.46	
PTR5	wsnp_Ex_c2887_5330426	2A	72.00	3.29	C	112	2.34	T	431	2.71	20.63	3.28	Yes
PTR5	wsnp_Ex_c12354_19711297	3A	65.99	3.25	C	462	2.67	A	81	2.40	14.92	2.45	
PTR5	wsnp_Ra_c44141_50623811	3A	66.97	3.1	G	93	2.42	A	450	2.67	17.13	2.57	Yes
PTR5	wsnp_Ex_c2920_5385184	3B	84.54	3.84	C	370	2.53	T	173	2.85	31.86	4.99	Yes
PTR5	wsnp_Ex_rep_c67468_66068960	***6A***	52.57	3.16	G	396	2.53	A	147	2.89	27.07	4.66	Yes
PTR5	wsnp_Ex_c17575_26301455	***6A***	53.02	3.29	C	394	2.53	T	149	2.90	27.44	4.93	
SB	wsnp_JD_c12281_12555386	Unk^D^	0	3.4	C	484	4.85	A	59	4.87	10.87	0.42	
SB	wsnp_Ku_c44362_51657973	Unk	0	4.95	G	513	4.83	A	30	5.20	5.52	0.18	
SB	wsnp_Ex_c24700_33953160	1B	37.18	3.39	C	33	4.65	T	510	4.86	6.08	1.21	Yes
SB	wsnp_JD_c8926_9848514	1B	37.25	3.39	G	33	4.65	A	510	4.86	6.08	1.21	
SB	wsnp_Ex_c15342_23592740	5A	76.51	3.32	C	58	5.05	T	485	4.83	10.68	0.22	
SB	wsnp_Ku_c17951_27138894	5A	76.51	3.65	G	500	4.83	A	43	5.10	7.92	0.14	
SB	wsnp_Ex_rep_c70120_69069789	5B	109.52	3.02	C	200	4.85	T	343	4.85	36.83	1.69	Yes
SB	wsnp_Ku_c50354_55979952	5B	146.88	3.67	C	380	4.89	T	163	4.76	30.02	5.80	Yes
SB	wsnp_Ku_c20701_30355248	5B	147.03	3.48	G	167	4.82	A	376	4.87	30.76	4.97	Yes
SB	wsnp_Ex_c15785_24157360	6B	90.36	3.39	G	505	4.87	A	38	4.66	7	1.73	Yes
SB	wsnp_Ex_c52527_56097039	***7B***	56.80	3.49	G	337	4.91	A	206	4.75	37.94	2.65	
SNB	wsnp_Ex_c23239_32477458	***2D***	173.84	3.91	C	59	2.85	T	484	3.59	10.87	11.07	Yes
SNB	wsnp_Ku_c9269_15583444	***2D***	173.84	5.31	G	57	2.73	A	486	3.60	10.5	12.28	Yes
SNB	wsnp_BE426620D_Ta_2_2	***2D***	175.64	3.33	C	93	3.07	T	450	3.60	17.13	8.98	
SNB	wsnp_CAP11_c318_261649	3A	82.55	3.03	G	366	3.68	A	177	3.15	32.6	8.72	Yes
SNB	wsnp_Ex_c5047_8963671	3A	99.60	3.35	C	191	3.19	T	352	3.68	35.17	8.87	
SNB	wsnp_Ku_c40334_48581010	***5B***	96.26	8.46	C	384	3.70	T	159	3.04	29.28	14.54	Yes
SNB	wsnp_Ku_c2185_4218722	***5B***	102.84	3.2	C	196	3.28	T	347	3.64	36.1	6.32	
SNB	wsnp_CAP12_c2547_1227972	***5B***	123.77	3.17	G	507	3.47	A	36	4.03	6.63	0.63	
STB	wsnp_Ex_c12220_19528388	3B	101.36	3.54	G	343	44.41	T	200	26.67	36.83	1.09	Yes
STB	wsnp_RFL_Contig4792_5787180	3B	163.73	4.84	G	148	52.53	A	395	32.46	27.26	12.84	Yes
STB	wsnp_CAP11_c59_99317	3B	163.73	4.53	G	150	52.15	A	393	32.50	27.62	12.54	
STB	wsnp_CAP11_c59_99769	3B	163.73	4.66	G	154	52.38	A	389	32.21	28.36	12.82	
STB	wsnp_Ex_c5744_10087758	6B	102.42	3.64	G	203	48.24	T	340	31.72	37.38	6.41	
STB	wsnp_Ex_rep_c106072_90285324	6B	102.42	3.57	C	211	47.79	T	332	31.59	38.86	6.99	Yes
STB	wsnp_JD_c646_966400	7B	40.62	3.33	G	147	48.26	A	396	33.98	27.07	3.74	Yes

A BLS  =  Bacterial leaf streak, PTR 1  =  *Pyrenophora tritici-repentis* race 1, PTR 5  =  *Pyrenophora tritici-repentis* race 5, SB  =  Spot blotch, SNB  =  Stagonospora nodorum blotch, and STB  =  Septoria tritici blotch, respectively.

B The bold and italicized genomic regions also were detected in association analysis using DArT markers (43–45). Septoria tritici-blotch (STB) was not included in association analysis using DArT markers.

C R-square calculated using simple regression.

D Chromosomal location is unknown.

**Table 5:** Gene annotation related to sequences of single nucleotide polymorphism (SNP) markers in the quantitative trait loci (QTL).

Disease^A^	Chr	Position (cM)	SNP marker	Gene symbol	Synonymous/non-synonymous	Amino acid change	Gene annotation
BLS	1A	82.03	wsnp_Ex_c44263_50363536	ATPAH1, PAH1	S		Lipin, N-terminal conserved region family protein, expressed
BLS	1A	85.09	wsnp_Ku_c23926_33870364		S		
BLS	5A	64.81	wsnp_Ex_c5998_10513766	0	NS	E/G	Chaperone DnaJ-domain superfamily protein
BLS	5D1cult	42.45	wsnp_Ex_rep_c67164_65655648	ACLA-3	NS	L/R	ATP-citrate lyase A-3
BLS	6B	120.91	wsnp_Ku_c11846_19263340	ATMAK10, MAK10	NS	L/S	MAK10 homologue
PTR1	2B	185.08	wsnp_Ex_rep_c67561_66189356	ACA8, AT-ACA8	S		Autoinhibited Ca2+ −ATPase, isoform 8, ATPase E1–E2 type family protein/haloacid dehalogenase-like hydrolase family protein
PTR1	4B	85.23	wsnp_Ex_rep_c67159_65649966	ABCC5, ATABCC5, ATMRP5, MRP5	NS	-/R	Multidrug resistance-associated protein 5
PTR1	7A	159.51	wsnp_Ku_c26118_36079171	0	NS	Y/H	Protein kinase superfamily protein with octicosapeptide/Phox/Bem1p domain
PTR5	2A	72.00	wsnp_Ex_c2887_5330426	0	NS	I/T	Glycosyl hydrolase family 10 protein/carbohydrate-binding domain-containing protein
PTR5	3A	66.97	wsnp_Ra_c44141_50623811	ESP4	NS	I/M	HEAT repeat-containing protein
PTR5	3B	84.54	wsnp_Ex_c2920_5385184	ATOXS3, OXS3	NS	L/S	Oxidative stress 3
PTR5	6A	52.57	wsnp_Ex_rep_c67468_66068960	ATBFRUCT1, ATCWINV1	S		Glycosyl hydrolases family 32 protein
SB	Unk^B^	0.00	wsnp_JD_c12281_12555386	iqd2	NS	C/W	IQ-domain 2
SB	1B	37.18	wsnp_Ex_c24700_33953160	SAG12	NS	Q/R	Cysteine protease 1 precursor, putative, expressed
SB	5A	76.51	wsnp_Ex_c15342_23592740	0	NS	K/R	NagB/RpiA/CoA transferase-like superfamily protein
SB	5B	109.52	wsnp_Ex_rep_c70120_69069789	ATSYTA, NTMC2T1.1, NTMC2TYPE1.1, SYT1, SYTA/ATSYTB, NTMC2T1.2, NTMC2TYPE1.2, SYT2, SYTB	NS	K/E	Synaptotagmin A/Calcium-dependent lipid-binding (CaLB domain) family protein
SB	5B	146.88	wsnp_Ku_c50354_55979952	ATSYTA, NTMC2T1.1, NTMC2TYPE1.1, SYT1, SYTA	S		Synaptotagmin A
SB	5B	147.03	wsnp_Ku_c20701_30355248	0	NS	L/S	O-fucosyltransferase family protein
SNB	2D	173.84	wsnp_Ex_c23239_32477458	CYL1, NAGLU	NS	K/E	α-N-acetylglucosaminidase family/NAGLU family
SNB	2D	173.84	wsnp_Ku_c9269_15583444	THY-1	NS	D/G	Thymidylate synthase 1
SNB	3A	99.60	wsnp_Ex_c5047_8963671		NS	S/P	Expressed protein
SNB	5B	96.26	wsnp_Ku_c40334_48581010	0	NS	C/R	DHHC-type zinc finger family protein
SNB	5B	123.77	wsnp_CAP12_c2547_1227972	ATCDC5, ATMYBCDC5, CDC5	NS	T/A	MYB family transcription factor, putative, expressed
STB	3B	101.36	wsnp_Ex_c12220_19528388	0	NS	S/A	Nucleotide-diphospho-sugar transferases super family protein
STB	3B	163.73	wsnp_RFL_Contig4792_5787180	CCB1	NS	L/R	Cofactor assembly of complex C
STB	3B	163.73	wsnp_CAP11_c59_99317	CCB1	S		Cofactor assembly of complex C
STB	6B	102.42	wsnp_Ex_c5744_10087758	0	NS	L/V	0
STB	6B	102.42	wsnp_Ex_rep_c106072_90285324	0	NS	-/W	0
STB	7B	40.62	wsnp_JD_c646_966400	0	NS	S/G	Transmembrane proteins 14C

A BLS  =  Bacterial leaf streak, PTR 1  =  *Pyrenophora tritici-repentis* race 1, PTR 5  =  *Pyrenophora tritici-repentis* race 5, SB  =  Spot blotch, SNB  =  Stagonospora nodorum blotch, and STB  =  Septoria tritici blotch, respectively.

S  =  Synonymous, NS  =  Non-synonymous.

B Chromosomal location is unknown.

**Table 6:** Summary of stepwise regression.

Disease^A^	No. of significant markers	Markers included in stepwise regression	Phenotypic variation explained together (as %)
BLS	8	4	14.28
PTR1	8	5	13.8
PTR5	6	4	13.21
SB	11	4	8.12
SNB	8	4	28.3
STB	7	4	19.49

A BLS  =  Bacterial leaf streak, PTR 1  =  *Pyrenophora tritici-repentis* race 1, PTR 5  =  *Pyrenophora tritici-repentis* race 5, SB  =  Spot blotch, SNB  =  Stagonospora nodorum blotch, and STB  =  Septoria tritici blotch, respectively.

Eight SNPs were significantly (p<0.001) associated with resistance to PTR race 1 and located on chromosomes 2B, 4B, and 7A (Table 4; Figure 3). The phenotypic variation explained by these SNPs ranged from 1.0 to 6.5% (Table 4). The phenotypic mean difference between the alleles for the significant SNPs ranged from 0.36 to 0.67. Of the eight significant SNPs identified, three were associated with a gene model (Table 5) with two being non-synonymous (chromosome 4B and 7A) and one being a synonymous change (chromosome 2B) (Table 5). Among the eight significant SNPs, five fit into a stepwise regression and explained 13.8% of the phenotypic variation (Table 6). These SNPs belong to five QTL regions and were present on chromosomes 2B, 4B and 7A. For PTR race 5, six SNPs were significantly (p<0.001) associated with resistance and located on chromosomes 2A, 3A, 3B, and 6A. The phenotypic variation ranged from 2.5 to 5% (Table 4; Figure 3) while the phenotypic mean difference between the alleles for the significant SNPs ranged from 0.22 to 0.4. Among the six significant SNPs detected, four were associated with a gene model (Table 5). Of these four changes, three were non-synonymous (chromosome 2A, 3A and 3B) and one was synonymous (chromosome 6A). For the six significant SNPs, four fit into a stepwise regression and explained 13.2% of the phenotypic variation (Table 6). These markers belong to four QTL regions and were present on chromosomes 2A, 3A, 3B, and 6A.

Eleven SNPs were associated with SB resistance and detected on chromosomes 1B, 5A, 5B, 6B and 7B (Table 4, Figure 3). The phenotypic variation explained by the eleven SNPs ranged from 0.1 to 5.8% (Table 4). The location of the QTL identified by SNP markers wsnp_JD_c12281_12555386 and wsnp_Ku_c44362_51657973 (*R^2^* ranged from 0.2 to 0.4) could not be assigned to a chromosome because the map location are unknown. The phenotypic mean difference between the alleles for the significant markers is ranged from 0.01 to 0.4. Of the eleven significant markers identified, six were associated with a gene model (Table 5). Of these six changes, five were non-synonymous on chromosome 1B, 5A, and 5B and one was synonymous located on chromosome 5B (Table 5). Among the 11 significant markers, four fit into a stepwise regression and explained 8.1% of the phenotypic variation (Table 6). These markers were located on four QTL regions on chromosomes 1B, 5B, and 6B.

In total, eight SNPs were significantly (p<0.001) associated with SNB resistance and detected on chromosomes 2D, 3A and 5B (Table 4; Figure 3). The phenotypic variation explained ranged from 0.01 to 14.5% (Table 4). The phenotypic mean difference between the alleles for the significant markers ranged from 0.38 to 0.90. Of the eight significant SNPs detected, five were associated with a gene model (Table 5) and were non-synonymous. Of the eight significant SNPs, four fit into a stepwise regression (Table 4) and explained 28.3% of the phenotypic variation (Table 6). These SNPs belong to three QTL regions.

Seven SNPs were significantly (p<0.001) associated with resistance to STB and detected on chromosomes 3B, 6B and 7B (Table 4, Figure 3). These belong to four QTL regions based on LD cutoff distance of 4 cM. The phenotypic variation explained by the seven SNP markers ranged from 1.1 to 12.8% (Table 4). The phenotypic mean difference between the alleles for the significant SNPs ranged from 14.98 to 20.95. Of the seven significant SNPs detected, six were associated with a gene model (Table 5). Of these six changes, five were non-synonymous on chromosome 3B, 6B and 7B and two changes were synonymous located on chromosome 3B (Table 5). Among seven significant SNPs, four fit into a stepwise regression and explained 19.5% of the phenotypic variation (Table 6). SNPs from four QTL regions on chromosomes 3B, 6B, and 7B were included in the stepwise regression model.

### Linkage disequilibrium and allelic combinations

**Figure 4:**
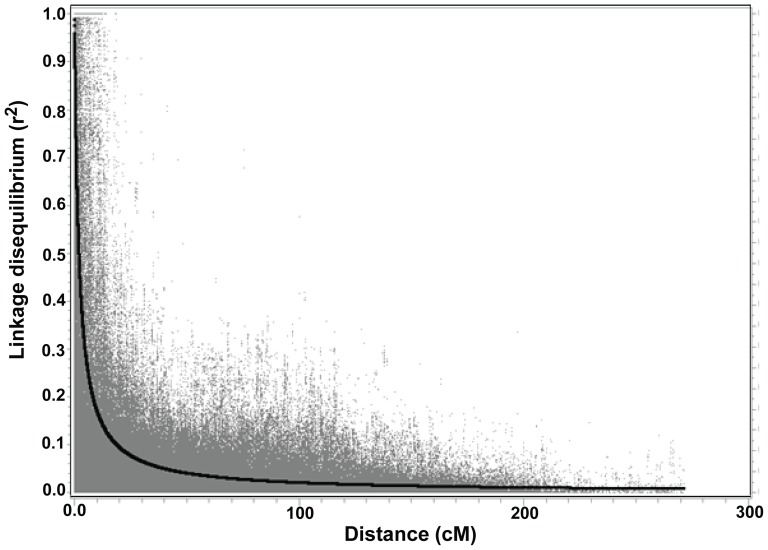
Genome-wide linkage disequilibrium (LD) decay plot for 528 spring wheat landraces based on 4,781 polymorphic single nucleotide polymorphism (SNP) markers. Linkage disequilibrium, measured as *R* ^2^, between pairs of polymorphic marker loci is plotted against the genetic distance (cM). Based on a LD cutoff of 0.7 (correlation is ±0.83) the critical LD was defined at a distance of <4 cM.

**Figure 5:**
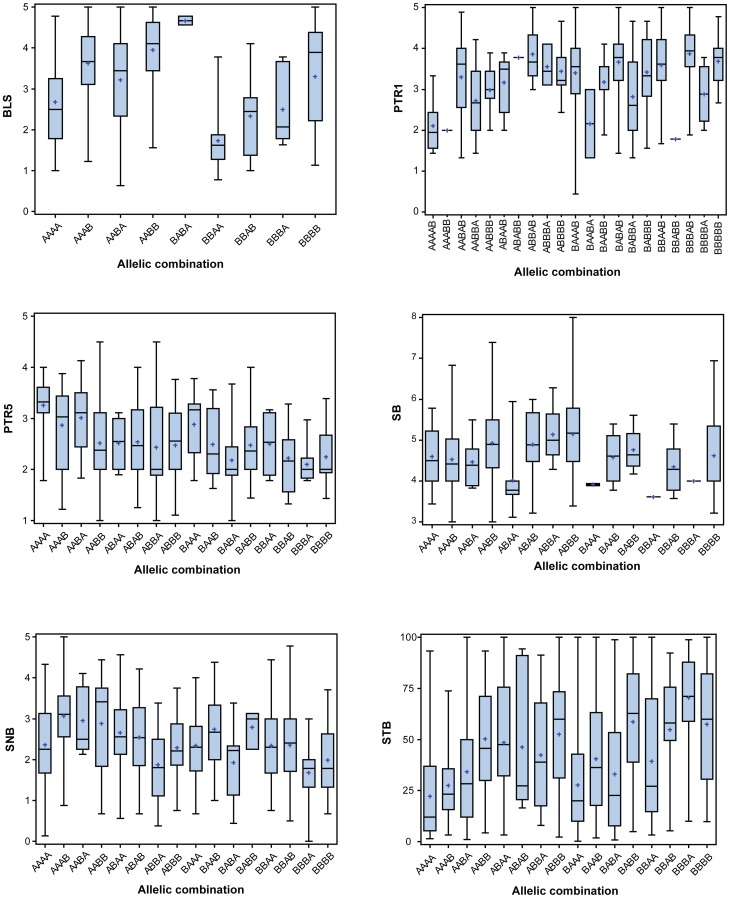
Box plots of the allelic combinations mean (Plus shape), 25th and 75^th^ percentile (colored box), median (center line in box), range of phenotype (in Whiskers).

Based on a LD cutoff of 0.7 (correlation is ±0.83), the critical LD was defined at a distance of <4 cM (Figure 4). Significant markers within a 4 cM interval can be defined as a single QTL. The numbers of allelic combinations for each disease ranged from 9 to 21 based on markers included in the stepwise regression (Table 7, Figure 5). Except for PTR race 5, all had at least one resistant allelic combination. Of the 528 spring wheat landraces analyzed, nearly 40% were susceptible to all leaf spot diseases and 45.4% were resistant to at least one disease (Table S3). Importantly, four wheat landraces were resistant to at least three leaf spot diseases (STB, SNB and BLS) and 65 landraces were resistant to at least two of the diseases.

**Table 7:** Allelic combinations based on the stepwise included markers.

Disease	Allelic combination	No. of observations	Minimum	Maximum	Mean	Std Dev^A^	Reaction
BLS	BBAA^B^	16	0.78	3.78	1.73	0.75	Resistant
BLS	BBAB	18	1.00	4.11	2.33	1.03	Susceptible
BLS	BBBA	6	1.63	3.78	2.50	0.97	Susceptible
BLS	AAAA	49	1.00	4.78	2.68	1.08	Susceptible
BLS	AABA	81	0.63	5.00	3.22	1.17	Susceptible
BLS	BBBB	13	1.13	5.00	3.31	1.35	Susceptible
BLS	AAAB	88	1.22	5.00	3.62	0.84	Susceptible
BLS	AABB	255	1.56	5.00	3.95	0.84	Susceptible
BLS	BABA	2	4.56	4.78	4.67	0.16	Susceptible
PTR1	BBABB	1	1.78	1.78	1.78	.	Resistant
PTR1	AAABB	1	2.00	2.00	2.00	.	Resistant
PTR1	AAAAB	6	1.44	3.33	2.11	0.69	Susceptible
PTR1	BAABA	2	1.33	3.00	2.17	1.18	Susceptible
PTR1	AABBA	25	1.44	4.22	2.72	0.93	Susceptible
PTR1	BABBA	18	1.33	4.67	2.83	1.11	Susceptible
PTR1	BBBBA	4	2.00	3.78	2.89	0.81	Susceptible
PTR1	AABBB	9	2.00	3.89	2.99	0.70	Susceptible
PTR1	ABAAB	6	2.00	3.89	3.17	0.76	Susceptible
PTR1	BAABB	9	1.89	4.11	3.17	0.77	Susceptible
PTR1	AABAB	80	1.33	4.89	3.30	0.99	Susceptible
PTR1	BAAAB	47	0.44	5.00	3.41	0.87	Susceptible
PTR1	BABBB	12	1.56	4.67	3.43	0.92	Susceptible
PTR1	ABBBB	5	2.44	4.67	3.44	0.83	Susceptible
PTR1	ABBBA	3	3.11	4.11	3.55	0.51	Susceptible
PTR1	BBAAB	20	1.67	5.00	3.59	0.90	Susceptible
PTR1	BABAB	178	1.44	5.00	3.67	0.79	Susceptible
PTR1	BBBBB	14	2.67	4.78	3.69	0.56	Susceptible
PTR1	ABABB	1	3.78	3.78	3.78	.	Susceptible
PTR1	ABBAB	15	3.00	5.00	3.86	0.63	Susceptible
PTR1	BBBAB	72	1.89	5.00	3.87	0.68	Susceptible
PTR5	BBBA	10	1.78	2.97	2.10	0.37	Susceptible
PTR5	BABA	13	1.00	3.67	2.18	0.76	Susceptible
PTR5	BBAB	14	1.33	3.28	2.22	0.68	Susceptible
PTR5	BBBB	18	1.43	3.39	2.25	0.59	Susceptible
PTR5	ABBA	6	1.00	4.50	2.44	1.23	Susceptible
PTR5	BABB	24	1.44	4.00	2.48	0.68	Susceptible
PTR5	ABBB	23	1.11	3.76	2.48	0.73	Susceptible
PTR5	BAAB	12	1.63	3.56	2.49	0.71	Susceptible
PTR5	BBAA	4	1.78	3.17	2.50	0.71	Susceptible
PTR5	ABAA	6	1.90	3.11	2.52	0.50	Susceptible
PTR5	AABB	230	1.00	4.50	2.52	0.75	Susceptible
PTR5	ABAB	10	1.25	4.00	2.54	0.85	Susceptible
PTR5	AAAB	54	1.22	3.88	2.86	0.71	Susceptible
PTR5	BAAA	13	1.78	3.78	2.88	0.63	Susceptible
PTR5	AABA	37	1.83	4.13	3.01	0.64	Susceptible
PTR5	AAAA	54	1.78	4.00	3.26	0.51	Susceptible
SB	BBAA	1	3.61	3.61	3.61	.	Resistant
SB	BAAA	2	3.89	3.94	3.92	0.04	Resistant
SB	BBBA	1	4.00	4.00	4.00	.	Resistant
SB	ABAA	7	3.11	5.94	4.02	0.90	Susceptible
SB	BBAB	6	3.57	5.39	4.35	0.69	Susceptible
SB	AABA	10	3.83	5.50	4.46	0.63	Susceptible
SB	AAAB	101	3.00	6.83	4.53	0.77	Susceptible
SB	BAAB	10	3.78	5.39	4.58	0.59	Susceptible
SB	AAAA	11	3.44	5.78	4.60	0.75	Susceptible
SB	BBBB	9	3.22	6.94	4.63	1.22	Susceptible
SB	BABB	4	4.17	5.61	4.77	0.61	Susceptible
SB	ABAB	22	3.22	6.00	4.89	0.79	Susceptible
SB	AABB	195	3.00	7.39	4.92	0.84	Susceptible
SB	ABBA	4	4.28	6.28	5.14	0.83	Susceptible
SB	ABBB	145	3.39	8.00	5.15	0.88	Susceptible
SNB	BBBA	35	0.00	3.00	1.68	0.66	Resistant
SNB	ABBA	30	0.38	3.38	1.87	0.81	Resistant
SNB	BABA	15	0.44	3.38	1.93	0.83	Resistant
SNB	BBBB	15	0.67	3.71	1.99	0.86	Resistant
SNB	ABBB	16	0.75	3.75	2.29	0.73	Resistant
SNB	BBAA	30	0.75	4.44	2.34	0.88	Resistant
SNB	BAAA	16	0.67	4.00	2.34	0.85	Resistant
SNB	BBAB	28	0.50	4.78	2.35	1.01	Resistant
SNB	AAAA	75	0.13	4.33	2.37	0.97	Resistant
SNB	ABAB	40	0.67	4.22	2.54	0.83	Resistant
SNB	ABAA	62	0.56	4.56	2.66	0.93	Resistant
SNB	BAAB	49	1.00	4.38	2.74	0.86	Resistant
SNB	BABB	3	2.25	3.13	2.79	0.48	Resistant
SNB	AABB	8	0.67	4.44	2.89	1.30	Resistant
SNB	AABA	5	2.13	4.11	2.95	0.92	Resistant
SNB	AAAB	101	0.88	5.00	3.06	0.80	Susceptible
STB	AAAA	63	1.43	93.33	22.30	23.05	Resistant
STB	AAAB	14	3.38	73.75	27.49	17.75	Resistant
STB	BAAA	139	0.25	100.00	27.81	22.53	Resistant
STB	BABA	35	0.75	98.89	33.06	30.23	Susceptible
STB	AABA	55	1.00	100.00	34.28	26.63	Susceptible
STB	BBAA	23	3.22	100.00	39.42	33.56	Susceptible
STB	BAAB	56	1.78	100.00	40.63	25.68	Susceptible
STB	ABBA	18	8.00	91.25	42.31	27.73	Susceptible
STB	ABAB	6	16.50	94.44	46.24	36.52	Susceptible
STB	ABAA	14	3.29	100.00	48.45	30.52	Susceptible
STB	AABB	13	4.38	93.33	50.30	29.73	Susceptible
STB	ABBB	13	2.33	100.00	52.69	27.76	Susceptible
STB	BBAB	10	5.22	92.22	54.73	29.42	Susceptible
STB	BBBB	18	9.89	100.00	57.49	28.66	Susceptible
STB	BABB	12	5.00	100.00	58.60	31.90	Susceptible
STB	BBBA	39	10.00	98.89	70.54	18.94	Susceptible

A Standard deviation.

B A and B refer to the alleles in the 9K SNP wheat chip.

Order of BLS markers - wsnp_Ex_c10596_17293363, wsnp_CAP11_rep_c4157_1965583, wsnp_Ex_c5998_10513766, wsnp_Ex_rep_c67164_65655648; Order of PTR1 markers - wsnp_BF473744B_Ta_2_2, wsnp_Ex_c19772_28771627, wsnp_Ex_rep_c67159_65649966, wsnp_Ex_c9971_16412345, wsnp_Ex_c9971_16412270; Order of PTR5 markers - wsnp_Ex_c2887_5330426, wsnp_Ra_c44141_50623811, wsnp_Ex_c2920_5385184, wsnp_Ex_rep_c67468_66068960; Order of SB markers - wsnp_Ex_c24700_33953160, wsnp_Ex_rep_c70120_69069789, wsnp_Ku_c50354_55979952, wsnp_Ex_c15785_24157360; Order of SNB markers-wsnp_Ex_c23239_32477458, wsnp_Ku_c9269_15583444, wsnp_CAP11_c318_261649, wsnp_Ku_c40334_48581010; Order of STB markers - wsnp_Ex_c12220_19528388,wsnp_RFL_Contig4792_5787180,snp_Ex_rep_c106072_90285324, wsnp_JD_c646_966400.

## Discussion

In spring wheat, few sources of broad-spectrum resistance to major leaf spot diseases are available. Due to this limitation, tremendous efforts have been made in the past decades to identify and introduce new sources of resistance from wild tetraploid wheat, such as emmer (*T. diccoccum*), Persian (*T. cathalicum*) and Polish (*T. polanicum*), and other wheat-alien species derivatives [69], [70], [71]. Recently, it has become feasible to rapidly test for thousands of SNP markers [39], [46]. In the present study, we analyzed association between disease resistance and SNPs from an association mapping panel of 528 spring wheat landraces. Our data indicate that spring wheat landraces exhibit considerable phenotypic and molecular variation, possibly due to the diverse genetic background of the accessions. We identified 32 SNPs significantly associated with loci conferring resistance to major leaf spot diseases. To validate broader applicability SNPs and GWAS, we also sought to verify resistances that were previously detected using DArT markers [43], [44],[45]. The higher marker density utilized in the present study enabled validation of previous findings [25], [26], [27]. Indeed, most of the loci detected previously by DArT markers also were identified by SNPs. Many of the SNPs significantly associated with QTL were found to be co-localized with candidate genes for plant defense and host plant resistance to several important diseases. This study provides a first step towards pyramiding resistance loci from these donors via MAS, which will enhance the genetic diversity for resistance in modern wheat germplasm and facilitate accelerated breeding to develop broad-spectrum resistance to manage leaf spot diseases of spring wheat.

The high-throughput SNP genotyping array and a high-density map developed previously have enabled GWAS to identify putative QTL associated with disease resistance [46]. In contrast to bi-parental mapping, association mapping provides us an excellent opportunity to analyze a larger pool of wheat accessions to uncover QTL. Furthermore, an association mapping panel with high MAFs, low LD, and limited population structure are ideal to perform association mapping analysis [49]. To investigate genetic structure, we performed a comprehensive GWAS to discover and localize QTL in spring wheat landraces of diverse geographic origin. Our data analysis supports hypothesis that spring wheat mapping panel did not have a strongly defined population structure and in addition the panel had a low LD, thus making it an excellent source for AM of multiple leaf spot diseases of wheat.

Population structure using structure analysis suggested two to six sub-populations. Previously, population structure was assessed for the same population using DArT markers [45], which were developed from coding regions. However, the SNPs tested here are from across wheat genome with various levels of selection pressure, presumably leading to a better picture of the population structure. When population structure is present, using the structure to control for spurious associations is imperative in association mapping analysis [72]. However, the relationship between individuals varied greatly, which might be due to unique selection pressures in each of the diverse environment where the accessions originated. These factors should be adjusted appropriately in an association analysis to control false positives [49].

One of the prerequisites for GWAS is LD, which is the non-random association of alleles at separate loci located on the same chromosome. The marker density needed for achieving a reasonable mapping resolution is highly related to the distance at which LD declines with genetic or physical distance. The amount of LD decay also varies with different crops. For example, LD decayed within 20 to 30 cM in rice [73], [74] and 10 to 40 cM in wheat [75], [76] based on different samples and marker systems used. If the LD distance is too large the QTL extends to 10 cM, making it difficult to identify the significant genes within the QTL region. Such large LD distance is possible if the population has narrow genetic diversity. In the present study, the extent of LD decay was about 4 cM. In general, if the LD is low, more markers are necessary. Although we found several SNPs associated with resistance to wheat leaf spot diseases, marker coverage was not distributed evenly across the genome, suggesting that the present study may have been unable to detect QTL in the genomic regions with low marker density.

We deployed 4,781 SNPs to perform GWAS and identified 48 significant SNPs associated with resistance to major leaf spot diseases of wheat. These constituted 32 QTL and explained the phenotypic variation (*R* ^2^) ranging from 0.6 to 14.5%. As expected, the phenotypic variation effect was low compared to previously reported QTL detected from bi-parental mapping [9], [10], [14], [25], [77]. The large differences in the explained phenotypic variations of QTL reported in linkage versus association mapping could be due to the number of recombinant events under study. A stepwise regression was used to find the subset of markers and QTL that can have a masking effect on minor effect markers and QTL [62], [78]. This procedure will limit the markers for MAS. With this approach, we were able to limit the number of loci to 25 markers and 24 QTL regions. Some of the QTL detected in this study may have already been previously identified in other studies. However, the positions cannot be related precisely due to the use of different linkage maps and markers for each of these studies.

A high level of diversity of wheat accessions can help us to better understand the genetics of resistance and to identify novel genomic regions linked with resistance genes. Due to low marker coverage of the D genome, we were unable to identify genetic regions within this genome associated with BLS, PTR 1, PTR 5, SB and STB disease resistance. In addition, none of the QTL identified were common among the different diseases.

Four QTL were identified for BLS resistance on chromosomes 1A, 5A, 5D, and 6B. Of these four QTL, those on 1A and 6B were also detected in association analysis using DArT markers [44]. The remaining QTL were mapped in novel genomic regions where no QTL were previously reported. The QTL responsible for PTR race 1 resistance were mapped to chromosomes 2B, 4B, and 7A. Some major and minor QTL regions on 2B and 7A were previously identified [14], [56]. The QTL on 2B also was detected in AM analysis using DArT markers [45]. Ptr ToxB toxin sensitivity gene *Tsc2* is located on chromosome 2B [14]. Similarly, another major QTL, *QTs-ksu-2B*, has also been mapped to chromosome 2B [79]. In addition, 2B has a QTL responsible for resistance to a novel PTR isolate [56]. Chromosome 7A, where the present study found the QTL responsible for resistance to PTR race 1, also has QTL responsible for resistance to novel PTR isolates [56]. QTL associated with resistance to PTR race 5 were identified on chromosomes 2A, 3A, 3B, and 6A. The genomic region 6A also was detected in a GWAS analysis using DArT markers [45]. Similarly, QTL identified in the genomic region 2A coincide with the host selective toxin insensitivity QTL *QTs.fcu-2A* [15], which conferred resistance to all known races of PTR tested. None of the other three QTL identified were mapped previously, and thus were considered novel. The present study further confirmed that the PTR-wheat pathosystem is complex and that targeting toxin insensitivity gene alone will not inevitably lead to PTR resistance [15], [17].

Of the five genomic regions (chromosomes 1B, 5A, 5B, 6B, and 7B) identified for SB resistance, 1B, 5B and 7B were mapped previously [18], [19], [79]. The genomic region 7B also was detected in a previous study [44]. Likewise, one minor QTL with phenotypic variation effect (R^2^) of 15.1% was detected on chromosome 1B [80] and one major QTL, *Qsb.bhu-5B*, was mapped on chromosome 5B [18], [19]. No previous evidence was observed for resistance to SB on chromosome 5A and thus this region appears to be a novel.

Three genomic regions (2D, 3A and 5B) had major and minor QTL responsible for SNB resistance [20], [22], [43]. The genomic regions 2D and 5B were detected previously [43]. The QTL on 2D and 5B were previously identified with the major genes *Snn2*, responsible for sensitivity to SnTox2, and *tsn1*, responsible for sensitivity to SnToxA [81], [82]. Three QTL responsible for resistance to STB were detected on 3B, 6B, and 7B, where no QTL have been detected previously. GWAS also was able to detect SNPs associated with QTL that were identified previously with DArT markers. For example, genomic regions 1A and 6B for BLS, 2B for PTR race 1, 6A for PTR race 5, 7B for SB, and 2D and 5B were detected using both SNP and DArT markers. However, some of the QTL detected in the previous studies were not found in this study. One possible explanation was that the mapping populations used to develop the consensus DArT and SNP maps were different, thus making it difficult to compare linkage maps. However, SNP markers were able to detect additional novel QTL which were not identified by DArT markers. This result could be expected since the SNP markers were more dispersed across the wheat genome compared to DArT markers, which tend to cluster and show low density in the centromeric regions and D genome of wheat [83]. SNP markers for GWAS may be more robust and cost-effective for QTL discovery than are DArT markers. Several major genes or QTL responsible for resistance to PTR race 1, SB, and SNB were detected, which also were previously identified from conventional bi-parental mapping.

GWAS can dissect the putative genes responsible for controlling phenotype [54], [84] and ann *in silico* approach was used to probe for such genes. The SNP marker sequences were blasted against database with coding regions of *Oryza sativa*, *Sorghum bicolor*, and *Brachypodium distachyon* and genes associated with plant disease resistance were identified. In addition, some of the gene models identified either have no known function or may not be involved in plant defense to pathogens. The sequences of SNPs associated with QTL for resistance to BLS show similarity to sequences coding for chaperone DnaJ-domain superfamily protein, ATP-citrate lyase A-3, and MAK10 homologue. The gene that encodes the chaperone DnaJ-domain super-family protein might play a critical role in biotic and abiotic stress response [85]. This gene was over-expressed in soybean revealing its vital role in cell death and disease resistance [85]. Another gene encodes ATP-citrate lyase and it may be involved in phytoalexin formation and was up-regulated in hot pepper leaves when challenged by a pathogen [86], [87].

Some QTL identified for resistance to PTR race 1 and PTR race 5 were found in the same genomic regions where known functional genes that are up-regulated in response to biotic or abiotic stress have been reported. For example, the SNP sequences that are linked to QTL responsible for PTR race 1 disease resistance are related to sequences coding for multidrug resistance-associated protein 5 and protein kinase superfamily protein with a octicosapeptide/Phox/Bem1p domain and are non-synonymous with these genes. Similarly, sequences for SNP markers that are non-synonymous and associated with resistance to PTR race 5 in the present study are related to sequences coding for glycosyl hydrolase family 10 protein/carbohydrate-binding domain-containing protein, a heat repeat-containing protein, and oxidative stress 3. For example, haloacid dehalogenase phosphatases were found in glycation repair by direct dephosphorylation of phosphoglycated proteins or DNA or by averting the intracellular concentrations of the phosphorylated aldoses from reaching toxic levels [88]. Other important genes that encodes Ca^2+^ -ATPase are directly or indirectly involved in several functions including processing of proteins in the secretary pathway, transport of Mn^2+^, and adaptation to salt stress [89]. The other gene that encodes a multidrug resistance-associated protein assisted in transporting the oxidized form of glutathione, a function essential in redox signaling activated by reactive oxygen species (ROS) in plant reactions to pathogen attack [90]. Yet another gene that encodes a protein kinase super-family protein that was associated with biotic and abiotic stress in plants [91], [92], [93]. A calcium dependent protein kinase has been reported to be the major component of innate immunity signaling pathways and some of the receptor-like protein kinases have been associated with plant defense responses.

The sequences from SNPs that are non-synonymous and are linked to QTL responsible for resistance to SB are related to cysteine protease 1 precursor, a NagB/RpiA/CoA transferase-like superfamily protein, a Calcium-dependent lipid-binding (CaLB domain) family protein, and an O-fucosyltransferase family protein. The roles of cysteine proteases and protease inhibitor genes in the regulation of programmed cell death in plants have been well-documented [94]. The calcium-dependent lipid-binding (CalB domain) family protein gene is concerned with transducing various stress signals to alter stress-regulated gene expression [95], [96].

The QTL responsible for resistance to SNB are possibly related to alpha-N-acetylglucosaminidase family/NAGLU family, thymidylate synthase 1, DHHC-type zinc finger family protein, MYB family transcription factor and all of these are non-synonymous changes. Of the several encoded genes for SNB, the zinc finger family protein plays a major role in plant disease resistance and has been shown to be highly unregulated and responsible for early defense responses against *E. amylovora* infection in apple [97]. The sequences of SNP markers linked to QTL conferring resistance to STB were found to be related to sequences for different disease resistance gene groups such as a nucleotide-diphospho-sugar transferases superfamily protein, a cofactor assembly of complex C cofactor assembly of complex C, and transmembrane proteins 14C. Of the genes encoding for STB, the transmembrane protein plays an important role in disease resistance [98]. In particular, the *Arabidopsis* NDR7 gene (contains two putative transmembrane domains) was essential for the expression of resistance to bacterial and fungal pathogens mediated by several R gene products [98]. Although we discovered several SNPs associated with novel QTL, functional analysis of the selected genes involved in host plant resistance needs further investigation.

### Implications for wheat disease resistance breeding

The genome-wide analysis of SNP markers in spring wheat landraces provided a basis for comprehensive analysis of QTL resistance to the major leaf spot diseases. We discovered potentially novel QTL and further confirmed a number of major and minor QTL detected in previous association analyses using DArT markers and bi-parental mapping approaches. Resistance to each leaf spot disease of wheat appears to be controlled by relatively high numbers of QTL. Pyramiding putative resistant alleles for resistance to several diseases had been successfully utilized in various crops via MAS [99], [100], [101]. The spring wheat landraces used in the present study harbor multiple putative resistant alleles, which can be useful for MAS breeding. We identified 32 QTL associated with resistance to the major foliar diseases of wheat and markers identified using stepwise regression and the allelic combinations would be good candidates for further marker validation work. For example, SNP markers wsnp_Ex_c10596_17293363, wsnp_CAP11_rep_c4157_1965583, wsnp_Ex_c5998_10513766 and wsnp_Ex_rep_c67164_65655648 can be used for MAS while developing wheat cultivars resistance to BLS. Similarly, wsnp_BF473744B_Ta_2_2, wsnp_Ex_c19772_28771627, wsnp_Ex_rep_c67159_65649966, wsnp_Ex_c9971_16412345, and wsnp_Ex_c9971_16412270 can be used for PTR 1, while wsnp_Ex_c2887_5330426, wsnp_Ra_c44141_50623811, wsnp_Ex_c2920_5385184 and wsnp_Ex_rep_c67468_66068960 can be used for PTR 5. Likewise, wsnp_Ex_c24700_33953160, wsnp_Ex_rep_c70120_69069789, wsnp_Ku_c50354_55979952, wsnp_Ku_c20701_30355248, and wsnp_Ex_c15785_24157360 can be used for SB, wsnp_Ex_c23239_32477458, wsnp_Ku_c9269_15583444, wsnp_CAP11_c318_261649, and wsnp_Ku_c40334_48581010 can be used for SNB, and markers wsnp_Ex_c12220_19528388, wsnp_RFL_Contig4792_5787180, wsnp_Ex_rep_c106072_90285324, and wsnp_JD_c646_966400 can be used for MAS while pyramiding QTL in wheat cultivars resistance to STB. One approach for validation would be to develop near-isogenic lines (NILs) in different genetic backgrounds via MAS backcrossing and evaluating them in multi-location field trials to confirm the efficacy of these QTL. Further, the broader effects of these QTL can be determined by testing NILs against multiple leaf spot pathogens. MAS breeding can be performed at an allelic level by combining several putative resistance QTL in a cultivar. In the present study, at least 15 spring wheat landraces had QTL for resistance to five of the six diseases tested. Based on the breeding target, wheat landraces from the present study could be selected as parents. For example, spring wheat landraces PI624606 and PI422235 were the most resistant accessions for majority of the leaf spot diseases tested in this study. These resistance sources could be crossed with commercial cultivars that are susceptible to various diseases. Progeny could be selected with both superior commercial traits and the markers for various disease resistant QTL. Finally, progeny with the highest number of putative resistance QTL could be further selected for testing disease resistance in multi-environments. This strategy may enable the development of cultivars with stable resistance to multiple leaf spot diseases of spring wheat.

## Supporting Information

Figure S1 **Comparison of QQ plots for different association models for major wheat leaf spot diseases.** Observed vs. expected P values are shown for (A) Bacterial leaf streak (BLS), (B) *Pyrenophora tritici-repentis race* 1 (PTR race 1) (C) *Pyrenophora tritici-repentis* race 5 (PTR race 5), (D) Spot blotch (SB), (E) Stagonospora nodorum blotch (SNB), (F) Septoria tritici blotch (STB) using four different models with different corrections of co-founding factors (see Materials and Methods). Based on MSD for the four regression models tested, a regression model that has only Kinship was considered best for resistance to PTR race 1, PTR race 5, and SNB and mixed model containing PC and Kinship were considered best for resistance to BLS, SB and STB.
(PDF)

Table S1 **Lists of wheat accessions along with their origin and disease reactions to multiple leaf spot diseases.** R and S determine if the genotype is resistant or susceptible based on the raw score of the wheat accessions.
(XLSX)

Table S2 **Spring wheat accessions and subpopulations (K = 6) identified using population structure analysis.**
(XLSX)

Table S3 **Resistant or susceptible reaction based on allelic combination mean.** Letter A and B refer to the alleles in the 9K SNP wheat chip.
(XLSX)
