# Supplementary material for: Genome-Wide Association Study Reveals Novel Quantitative Trait Loci Associated with Resistance to Multiple Leaf Spot Diseases of Spring Wheat
Source: PLoS One. 2014 Sep 30;9(9):e108179. doi: 10.1371/journal.pone.0108179 (PMC4182470; doi:10.1371/journal.pone.0108179)
Supplement: Figure S1 — Comparison of QQ plots for different association models for major wheat leaf spot diseases. Observed vs. expected P values are shown for (A) Bacterial leaf streak (BLS), (B) Pyrenophora tritici-repentis race 1 (PTR race 1) (C) Pyrenophora tritici-repentis race 5 (PTR race 5), (D) Spot blotch (SB), (E) Stagonospora nodorum blotch (SNB), (F) Septoria tritici blotch (STB) using four different models with different corrections of co-founding factors (see Materials and Methods). Based on MSD for the four regression models tested, a regression model that has only Kinship was considered best for resistance to PTR race 1, PTR race 5, and SNB and mixed model containing PC and Kinship were considered best for resistance to BLS, SB and STB. (PDF) [file pone.0108179.s001.pdf]

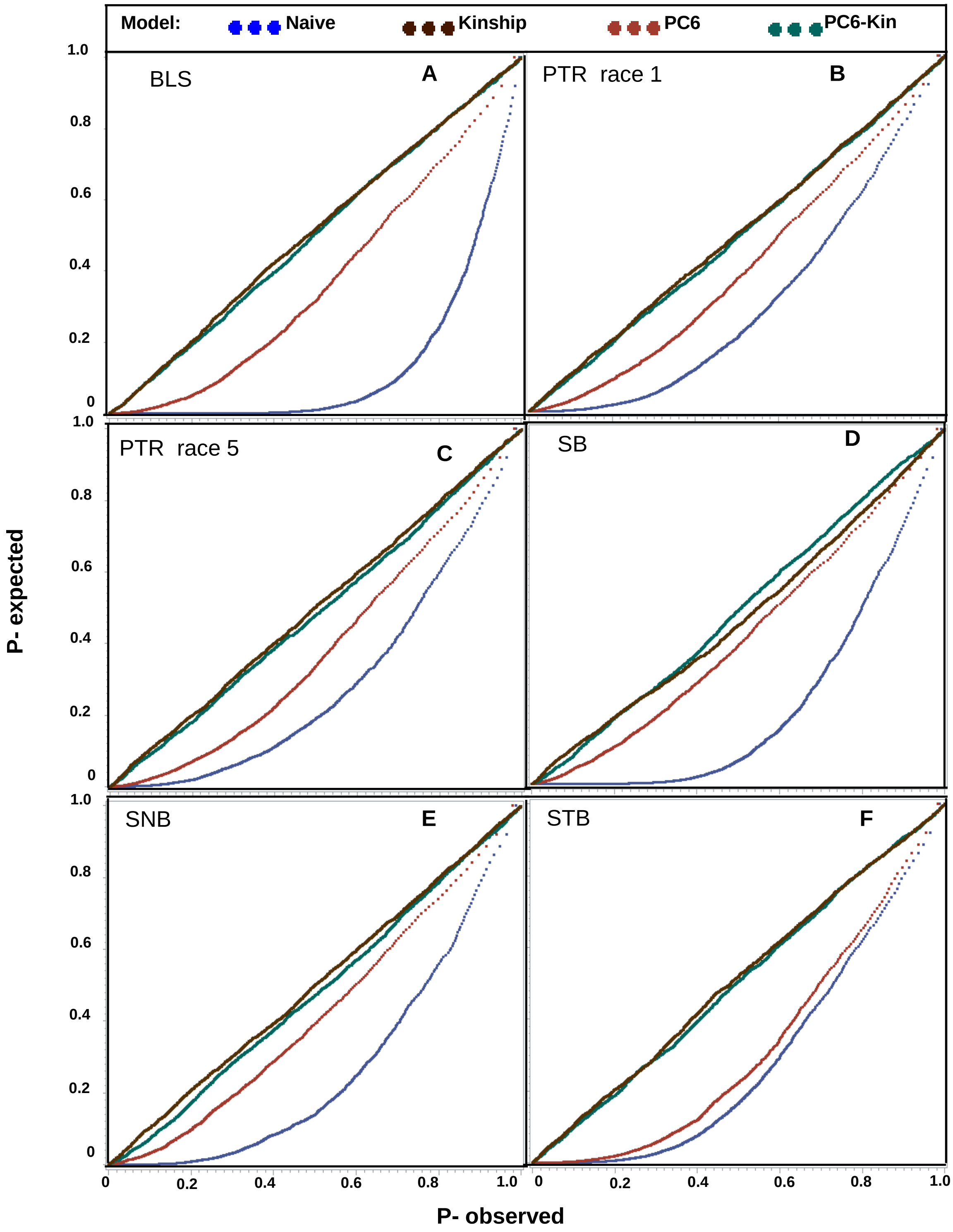

Fig. S1. Comparison of QQ plots for different association models for major wheat leaf spot diseases. Observed vs. expected P values are shown for (A) Bacterial leaf streak (BLS), (B) *Pyrenophora tritici-repentis race* 1 (PTR 1) (C) *Pyrenophora tritici-repentis* race 5 (PTR 5), (D) Spot blotch (SB), (E) Stagonospora nodorum blotch (SNB), (F) Septoria tritici blotch (STB) using four different models with different corrections of co-founding factors (Materials and Methods). Based on MSD for the four regression models tested, regression model that has only Kinship was considered best for PTR race 1, PTR race 5 and SNB disease trait and mixed model containing PC and Kinship were considered best for BLS, SB and STB.
